# Visual Pathways Involvement in Friedreich’s Ataxia Patients Without Macular Impairment

**DOI:** 10.3390/jcm15156097

**Published:** 2026-08-05

**Authors:** Vincenzo Parisi, Lucilla Barbano, Antonio Di Renzo, Carmen Dell’Aquila, Mattia D’Andrea, Angela Maria Castelluzzo, Gaspare Colacino, Valeria Gioiosa, Gianluca Coppola, Carlo Casali, Lucia Ziccardi

**Affiliations:** 1 IRCCS—Fondazione Bietti, Via Livenza 3, 00198 Rome, Italy; vincenzo.parisi@fondazionebietti.it (V.P.); antonio.direnzo@fondazionebietti.it (A.D.R.); carmen.dellaquila@fondazionebietti.it (C.D.); lucia.ziccardi@fondazionebietti.it (L.Z.); 2 Departmental Faculty of Medicine, UniCamillus-Saint Camillus International University of Health Sciences, 00131 Rome, Italy; 3 Department of Sense Organs, Faculty of Medicine and Dentistry, Sapienza University of Rome, Viale del Policlinico 155, 00161 Rome, Italy; mattia.dandrea@uniroma1.it; 4 Department of Medicine and Health Sciences “V. Tiberio”, University of Molise, Via F. De Sanctis 1, 86100 Campobasso, Italy; castelluzzouni@gmail.com; 5 UOC Oftalmologia—San Giovanni Evangelista Hospital Tivoli, Via Antonio Parrozzani, 3 00019 Tivoli, Italy; colacinogaspare@gmail.com; 6 Department of Medical and Surgical Sciences and Biotechnologies, Sapienza University of Rome, Corso della Repubblica 79, 04100 Latina, Italy; valeria.gioiosa@uniroma1.it (V.G.); gianluca.coppola@uniroma1.it (G.C.); carlo.casali@uniroma1.it (C.C.); 7 Neurogenetics Unit, Reference Center for Neurogenetic Disorders (BRAIN-TEAM Network), INCIA, CNRS UMR 5287, Bordeaux University, 33000 Bordeaux, France

**Keywords:** friedreich, ataxia, mfERG, PERG, VEP, OCT

## Abstract

**Objectives**: Visual Evoked Potential (VEP) abnormalities in Friedreich Ataxia (FA) may result from dysfunction of retinal ganglion cells (RGC), post-retinal visual pathways, or both. Therefore, we aimed to clarify whether in Friedreich Ataxia (FA), abnormal neural conduction is due to dysfunctional RGC, impaired conduction along the post-retinal visual pathways, or both. The secondary aim of the study was to clarify the relationship between a possible RGC and/or post-retinal dysfunction and the patient’s age, age at disease onset, and disease duration. **Method**: Fourteen FA patients (14 eyes; mean age 38.64 ± 8.21 years) without functional or structural macular abnormalities, confirmed by multifocal electroretinogram (mfERG) and spectral domain–optical coherence tomography (SD-OCT), and 20 age-matched healthy controls were enrolled. Patients were classified as late-onset (FA1, disease onset > 25 years) or early-onset (FA2, disease onset < 25 years). All participants underwent comprehensive ophthalmological and neurological evaluations. RGC function and visual pathway conduction were assessed through simultaneous pattern electroretinogram (PERG) and VEP recordings using 60′ and 15′ checkerboard stimuli. Retino-cortical time (RCT) was calculated as the difference between VEP P100 and PERG P50 implicit times. Statistical analyses included a general linear model. **Results**: 60′ PERG amplitude (A) was significantly (*p* < 0.01) reduced in the FA group compared to the Control group. FA patients showed significantly delayed 60′ and 15′ VEP ITs and significantly increased 60′ and 15′ RCT values, particularly in the FA1 group. In late-onset patients, no significant relationships were found between age at onset of disease and all electrophysiological parameters (60′ and 15′ VEP and PERG ITs and As and RCTs). **Conclusions**: In our selected FA, RCG function and neural conduction along the post-retinal visual pathways are impaired. The abnormal neuronal conduction is greater in late-onset FA patients and is not related to patients’ age and the age at the onset of the disease. The duration of the disease has a slight effect on the post-retinal neural conduction on small optic nerve fibers.

## 1. Introduction

Approximately 73% of patients with Friedreich Ataxia (FA) may present abnormalities in the visual nervous system with or without symptoms [1].

The function of the whole visual pathway (from the retinal photoreceptors to the occipital cerebral cortex) can be objectively assessed using Visual Evoked Potentials (VEP) recordings [2,3,4,5,6,7,8,9,10,11], but the VEP bioelectrical responses can be influenced by retinal ganglion cells (RGC) or macular impairment [12,13]. 

By using simultaneous recordings of VEP and pattern electroretinogram (PERG, which assesses RGC function [14,15,16,17,18]), it is possible to derive an electrophysiological index of the post-retinal neural conduction, named Retino-Cortical Time (RCT) [19]. Abnormal RCT in patients with demyelinating diseases [19], but also in patients with glaucoma [12], was found, suggesting a dysfunction in the post-retinal visual pathways.

The function of the outer retinal layers located in the macular area can be assessed using multifocal electroretinogram (mfERG) [20,21,22,23,24,25] recordings. Abnormal mfERG responses in patients with neurodegenerative diseases were observed [26,27]. 

At present, it is known that VEP abnormalities, suggesting a dysfunction of the neural conduction along the whole visual pathway, can be found in FA patients [28,29,30,31,32,33,34], and abnormal mfERG responses can be observed only in FA asymptomatic carriers [35], thus suggesting an early dysfunction of the macular pre-ganglionic elements.

However, it is unclear whether the above-mentioned VEP abnormalities [14,15,16,17,18,19,20,21,22,23,24,25,26,27,28,29,30,31,32,33,34] could be ascribed to a dysfunction occurring at the level of the retinal structures (macular outer retina or RGC) and/or at the level of the post-retinal visual pathways. 

All this constitutes the rationale for designing this cohort study, in which our aim was to clarify the source of the post-retinal visual pathways impairment. To exclude the possible macular contribution to the VEP impairment, FA enrolled patients who were exclusively those without macular morpho-functional involvement, established by normal mfERG responses and normal OCT scans.

In addition, the secondary objective of the study was to assess, in FA, whether a possible RGC and/or post-retinal dysfunction could be related to patients’ age, age at disease onset, and duration of disease.

## 2. Materials and Methods

### 2.1. Participants 

This observational prospective case series study has been performed at the Clinical and Research Centre of Neurophthalmology, Genetic and Rare Diseases of IRCCS Fondazione Bietti ETS in Rome on FA subjects followed and referred by the Department of Medical and Surgical Science and Biotechnologies, Sapienza University of Rome between October 2023 and June 2026.

The study protocol adhered to the Declaration of Helsinki and was approved by the local Ethics Committee (Comitato Etico Territoriale Lazio Area 5, protocol number 23/FB/23, approved on 6 September 2023). All patients were informed of the study outcomes and procedures and gave written informed consent prior to participation.

Fourteen FA patients harboring biallelic GAA triplet repeats in the first intron of the *FXN* gene in the typical range between 600–1300 (mean 791.62 ± SD 131.24) were selected from a large number of FA patients (49 patients) based on the below-mentioned exclusion criteria. Twenty healthy subjects without visual and neurological diseases served as controls.

All participants (FA and controls) underwent a complete neurological and ophthalmological examination comprising functional and morphological assessments of the retina, the macula, the RGC and the visual pathways.

Although few clinical studies have been conducted on FA subjects, limited extrapolated data report the absence of any symptoms but the susceptibility to develop diabetes [36]. Since it is known that diabetes may affect macular [37], RGC [38,39], and optic nerve function [39], we ruled out a priori all FA patients with a previous history of diabetes and with HbA1c level > 6.5%. 

Moreover, since several pathologies may induce changes in macular morpho-functional condition [40,41,42,43,44,45,46], studied eyes were selected on the basis of the following exclusion criteria: absence of target fixation placed on the monitor screen during the electrophysiological recordings, presence of lens opacity with a severity grade > 1.5 for nuclear, cortical and posterior subcapsular opacities according to AREDS Clinical Lens Opacity Standard Photographs System [47], ocular hypertension or glaucoma, diabetic retinopathy, drusen and age-related macular degeneration, nystagmus, history of uveitis and optic neuritis; eyes from patients with systemic diseases such as systemic hypertension, diabetes, connective tissue diseases were also excluded. All enrolled eyes were phakic, and the refractive error was between ±1.50 equivalent spherical diopters.

Based on the main aim of the study, another important exclusion criteria for FA eyes was the absence of binocular macular dysfunction assessed using mfERG recordings (see below Section 2.4 and Section 3.1 and Table 1 and Table 2) or binocular macular morphological impairment (such as macular edema, epiretinal membrane or any disruption at the level of ellipsoid zone or Retinal Pigment Epithelium) assessed using Spectral Domain–Optical Coherence Tomography (SD-OCT) [see below Section 2.4].

Fourteen selected FA patients (9 females and 5 males, mean age: 38.64 ± 8.21 years (age range: 18–48 years)) were enrolled in the study. For each FA patient, the age at disease onset and the duration of the disease, based on the onset of neurological symptoms (see below Section 2.2), were also considered. For each FA patient, the right eye was considered in the study. 

Based on the age at the onset of the disease (see Section 2.2), FA patients were divided into two groups:

(1) Group FA1: 7 late-onset FA patients with onset of symptoms after 25 years of age (2 males and 5 females, mean age: 39.42 ± 7.87 years), providing 7 right eyes;
(2) Group FA2: 7 early-onset FA patients with onset of symptoms during childhood or before 25 years of age (4 females and 3 males, mean age: 37.86 ± 9.08 years), providing 7 right eyes.

Control healthy subjects, without any visual or neurological diseases, were a total of 20 (15 females and 5 males, mean age: 33.15 ± 8.37 years, range 18–45 years), providing 20 right eyes with normal (0.0 LogMAR) Best-Corrected Visual Acuity (BCVA, see below Section 2.3).

### 2.2. Neurological and Non-Neurological Examination 

All FA subjects included in the study showed clinical neurological manifestations typical of FA such as cerebellar ataxia, movement disorders, pyramidal signs, peripheral nerve involvement, dysarthria, and hearing impairment, as well as non-neurological involvement including cardiomyopathy, kyphoscoliosis, and foot deformities. In all FA patients, the disease onset was considered to subdivide the group. For instance, early-onset FA was defined when symptoms onset during childhood or before 25 years of age, and late-onset FA was defined when symptoms onset after 25 years of age. The disease severity was assessed using the Scale for the Assessment and Rating of Ataxia (SARA) and ranged from 5/40 to 13/40 in the FA1 group and from 18/40 to 32/40 in the FA2 group.

HbA1c levels, assessed using high-performance liquid chromatography, were <6.5% in all enrolled FA patients. Where available, the number of GAA repeats was retrieved. It is worth noting that all patients included in the study carried biallelic GAA expansions; none showed compound heterozygosity consisting of a GAA expansion on one allele and a point mutation or deletion on the other allele. 

### 2.3. Visual Acuity Assessment

BCVA was evaluated by administering the modified self-illuminated Early Treatment Diabetic Retinopathy Study (ETDRS) charts (Lighthouse, Low Vision Products, Long Island City, NY, USA) at a distance of 4 m. BCVA was measured as LogMAR. 

### 2.4. Macular Morpho—Functional Examination

In FA and Control eyes, the macular morphological condition was evaluated by SD-OCT (Heidelberg Eye Explorer, Heidelberg Engineering, Heidelberg, Germany) images according to our previously published method [48].

The macular function was assessed using mfERG recordings using the Diagnosys LTD system (Diagnosys UK, LTD; Histon, Cambridge, UK), according to our previously published method [34] following the 2011 International Society for Clinical Electrophysiology of Vision (ISCEV) standards [20].

For the mfERG recordings, the multifocal stimulus, consisting of 61 scaled hexagons, was displayed on a high-resolution, black-and-white 32” LCD monitor (size, 69 cm width and 38 cm height) with a frame rate of 75 Hz. The array of hexagons subtended 50° of visual field (25° radius from the fixation point to edge of display). Each hexagon was independently alternated between black (1 cd/m^2^) and white (200 cd/m^2^) according to a binary m sequence. 

In all subjects, mfERG was binocularly recorded after pupil dilation (1% Tropicamide) to a diameter of 7 to 8 mm. The cornea was anesthetized with Benoxinate 0.4% eye drops. For all recordings, an active Dawson Trick Litzkow bipolar contact electrode, a reference electrode (Ag/AgCl skin electrode placed on the corresponding outer canthi), and a small Ag/AgCl skin ground electrode, placed in the center of the forehead, were used. Interelectrode resistance was lower than 3 KOhms. The signal was filtered (band pass 3–100 Hz) by the Espion system. After automatic rejection of artifacts, the first-order kernel response was considered. In the analysis of the mfERG, the averaged response amplitude density (RAD) was measured in nanoVolt/degree^2^ (ηV/degree^2^) as described in our previous works [48].

The mfERG responses were analyzed using the Ring analysis according to the previously published method [48].

### 2.5. RGC and Visual Pathways Function Examination

PERG and VEP were simultaneously recorded by using the Retimax Advanced Plus system (CSO, Florence, Italy), in accordance with previously described methodologies [12].

Visual stimulation was monocular, with the fellow eye occluded. Stimuli consisted of high-contrast (80%) checkerboard patterns presented on a monitor with a mean luminance of 110 cd/m^2^, reversing at 2 Hz. At a viewing distance of 114 cm, check sizes subtended either 60′ or 15′ of arc, as recommended by ISCEV standards [49] to preferentially stimulate large and small axons of the optic nerve, respectively. The monitor subtended a total visual angle of 23°, and a central fixation target (approximately 0.5°) was provided. All patients confirmed that the fixation target was clearly visible.

PERG signals were recorded via Ag/AgCl skin electrodes placed on the lower eyelid. A bipolar configuration was used, with the active electrode on the stimulated eye and the reference electrode on the patched eye. The P50 and N95 components were identified, and the P50 implicit time [PERG IT measured in milliseconds (msec)] and peak-to-peak P50–N95 amplitude [PERG A measured in microvolt (µV)] were measured.

For VEP responses, the N75 and P100 components were analyzed; the P100 VEP IT and peak-to-peak N75-P00 VEP A were measured. The RCT was calculated as the difference between VEP P100 and PERG P50 ITs [12].

Each recording session included a minimum of two and a maximum of six repeated acquisitions to verify waveform reproducibility. Based on previous findings [35], intra-individual variability was estimated as ±2 msec for VEP IT and ±0.18 µV for PERG A. Two recordings were considered reproducible if the difference fell within these ranges. When necessary, additional recordings were performed, never exceeding six per session. In addition, the two reproducible recordings provided the data for the test-retest intra- and inter-individual variability of the entire cohort of enrolled patients.

For statistical analysis, the averaged values of PERG and VEP parameters from reproducible traces were considered.

Signal-to-noise ratio (SNR) for PERG and VEP responses was assessed using previously established methods [12]. Only recordings with SNR values greater than 2 were included in the analysis.

### 2.6. Statistical Analysis 

Sample size was calculated by using preliminary groups of 5 participants, respectively, for FA and controls. 

The size evaluation is based on the following 15′ VEP IT values: 104.50 ± 5.63 msec for controls and 115.23 ± 8.95 msec for FA with α = 5% (type 1 error) and power = 80% (β = 20%), giving us 9 participants for each group.

Moreover, another sample size evaluation was performed to calculate the number of participants for FA 1 and FA 2 groups, again based on 15′ VEP IT.

Values are as follows: 123.61 ± 8.78 for the FA 1 group and 109.72 ± 8.52 for the FA 2 group, providing 7 observations for each group.

Normality tests were performed for each group’s parameter using Anderson-Darling and Kolmogorov-Smirnov methods.

Groups’ descriptive statistics were evaluated as mean and SD.

General linear model (GLM) was developed considering each electrophysiological parameter as dependent variables, subject’s age and disease duration as covariates, gender and groups (controls, patients: FA1 and FA2) as factors, to assess group differences, if any. 

Again, for each parameter, GLM was developed as shown above, considering patients’ Groups combined (FA1 + FA2 = FA group).

A *p*-value of 0.01 was considered statistically significant for group comparisons corrected for the number of tested parameters (Bonferroni: 0.05/5 = 0.01).

Lastly, GLM was developed for each parameter: 60′ and 15′ VEP ITs, 60′ and 15′ RCT, and BCVA as dependent variables, age, age at the onset of disease, and disease duration as covariates, and gender as a factor to assess the relationship between dependent variables and covariates, if any.

A *p*-value of 0.01 was considered statistically significant, corrected for the number of relationships assessed for each parameter (60′ and 15′ VEP ITs, 60′ and 15′ RCT, and BCVA: 0.05/5 = 0.01).

SPSS (version 25) software was used for statistical analysis.

## 3. Results

**Figure 1:**
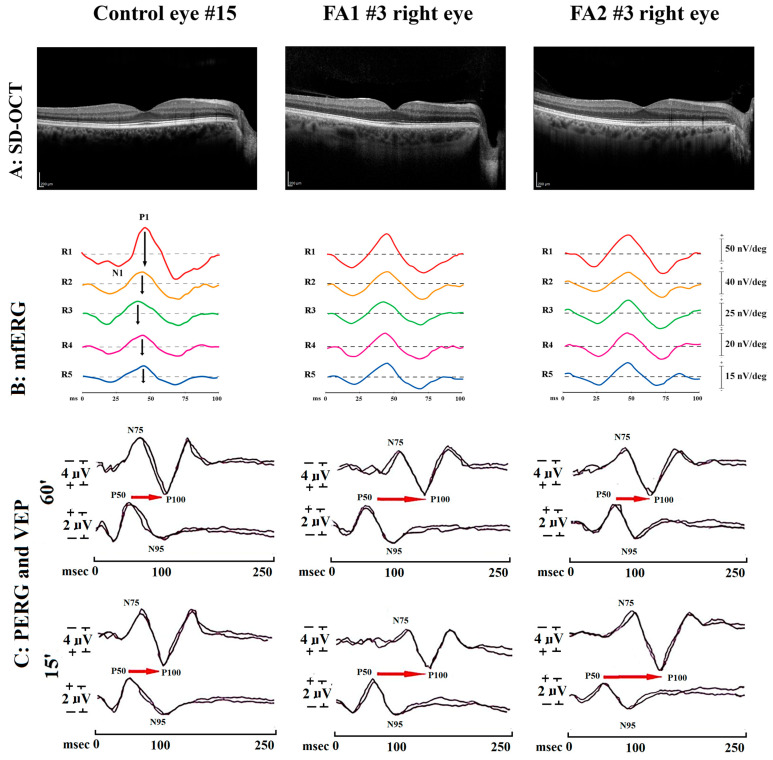
Examples of spectral domain–optical coherence tomography (SD-OCT) assessment. (**A**) Multifocal electroretinogram (mfERG) recordings (**B**), simultaneous recording of 15′ and 60′ pattern electroretinogram (PERG) and Visual Evoked Potentials (VEP) (**C**) from eyes of one representative control subject, of one late-onset (age at the onset of disease > 25 years) and of one early-onset (age at the onset of disease < 25 years) Friedreich Ataxia patients (FA1#3 right eye and FA2#3 right eye, respectively). (**A**) by OCT scans, no morphological abnormalities were observed in the inner or outer retinal layers in Control and in FA eyes. (**B**) in Control eye, mfERG traces showed normal the N1-P1 (↕) response amplitude density (RAD, measured in nanoVolt/degree^2^—nV/deg^2^); RADs were measured in five concentric annular retinal regions (rings) centered on the fovea: from 0 to 5 degrees (ring 1, R1, in red), from 5 to 10 degrees (ring 2, R2, in orange), from 10 to 15 degrees (ring 3, R3, in green), from 15 to 20 degrees (ring 4, R4, in purple) and from 20 to 25 degrees (ring 5, R5, in blue). In all Rings, RAD values detected in Control and in FA eyes were similar, thus identifying normal macular function. (**C**) 60′ and 15′ refers to check edges subtending 60 min (60′) and 15 min (15′) of the visual angle for PERG and VEP visual stimuli; N75 and P100 refer to the first negative and the first positive peak of VEP recordings (the implicit time of P100 and the peak-to-peak N75-P100 amplitude were considered); P50 and N95 refer to the first positive and the second negative peak of PERG recordings (the implicit time of P50 and the peak-to-peak P50-N95 amplitude were considered); the Retino-cortical Time (RCT), indicated by the red arrow, is the difference between VEP P100 and PERG P50 implicit times; msec = milliseconds. FA eyes and the control eye showed similar PERG P50-N95 amplitudes and P50 implicit times; VEP P100 implicit times and RCT were increased in the FA1 eye when compared to the control and FA2 ones.

Figure 1 shows illustrative examples of macular SD-OCT scans and mfERG, PERG, and VEP traces performed in one Control eye (Control eye#15) and in two Friedreich’s ataxia eyes with late-onset or early-onset disease (FA1 #3 right eye and FA2 #3 right eye, respectively).

Table 1 reports the individual values of patients’ age, duration of disease, age of onset of the disease, BCVA, mfERG, PERG and VEP parameters observed in late-onset and early-onset Friedreich’s ataxia patients.

### 3.1. mfERG Data

In the FA group, as shown in Table 2, the group factor was not significant (*p* > 0.01) for each considered R1-R5 RADs.

Again, as shown in Table 3, the group factor was not significant (*p* > 0.01) for each R1-R5 RADs assessed in Controls, FA1 and FA2 Groups.

### 3.2. VEP Data 

As shown in Table 2, in the FA group, mean values of P100 ITs were significantly increased (*p* < 0.001) with respect to those of the control group for both 60′ and 15′ of visual stimuli, and a significant (*p* < 0.001) reduction of 60′ and 15′ N75-P100 As was found.

Group factor (2-level factor: FA group and control) was significant for 60′ and 15′VEP ITs and 60′ and 15′ As. 

Table 3 shows a significant (*p* < 0.001) group factor (3-level factor: control, FA1 and FA2 groups) for 60′ and 15′ P100 ITs and N75 P100 As.

### 3.3. PERG Data

As reported in Table 2, group factors (controls and FA grouped together) showed significant 60′ PERG A, whereas not significant (*p* > 0.01) 15′ PERG A and 60′ and 15′ PERG IT were not found.

Considering the patients’ group separated into FA1 and FA2, group factors were not statistically significant (*p* > 0.01) for each PERG parameter (see Table 3).

### 3.4. Retino-Cortical Time Data

Group factor (FA group and control) was significant (*p* < 0.001) for 60′ and 15′ RCT, as shown in Table 2.

Table 3 shows a significant (*p* < 0.001) group factor (Control, FA1 and FA2 Groups) for 60′ and 15′ RCT.

### 3.5. Best-Corrected Visual Acuity (BCVA)

In the FA group, mean BCVA and SD values were 0.225 ± 0.261 LogMAR; GLM showed a significant (F = 72.43, *p* < 0.001) group factor (control 0.00 ± 0.00 LogMAR).

Again, GLM showed a significant group factor (F = 99.17, *p* < 0.001) when FA was sub-grouped into FA1 and FA2 groups. In the FA1 group, mean BCVA and SD values were 0.429 ± 0.16 LogMAR, whereas in the FA2 group, they were 0.00 ± 0.00 LogMAR. Again, the group factor was statistically significant.

### 3.6. Relationships Between VEP and RCT Data and Age, Age at Onset of the Disease, Duration of Disease

Mean age at onset of the disease was in the FA group of 23.86 ± 10.88 years (range 11–41 years), in the FA1 group of 33.28 ± 6.77 years (range 25–41 years), and in the FA2 group of 14.43 ± 1.81 years (range 11–16 years). Therefore, mean age at the onset of the disease was significantly (t = 6.76, *p* < 0.001) higher in the FA1 group when compared to the FA2 group.

Mean duration of the disease was 14.57 ± 10.27 years (range 2–31 years) in the FA1 group, 6.14 ± 1.86 years (range 2–7 years) in the FA2 group, and 23.00 ± 7.70 years (range 7–31 years). Therefore, the mean duration of the disease was significantly (t = −5.63, *p* = 0.001) higher in FA2 when compared with that of the FA1 group.

In Table 4, for FA1 and FA2 Groups, GLM was developed using 60′ and 15′ VEP ITs and 60′ and 15′ RCTs as dependent variables separately and age, age at the onset of the disease, and duration of disease as covariates to assess each covariate effect, if any. 

GLM analysis showed no significant relationships (*p* < 0.01) between 60′ and 15′ VEPs and RCTs and each covariate in FA1 and FA2 groups. 

By applying Bonferroni’s correction for multiple comparisons, the relationships between 60′ VEP IT and duration of the disease in the FA2 group, and between 15′ RCT and duration of the disease for the FA1 group were not confirmed (*p* > 0.01).

## 4. Discussion

The aim of our study was to evaluate, in FA patients, the neural conduction along the whole visual pathways (by VEP recording) and to assess whether potential VEP abnormalities should be due to RGC dysfunction (assessed using PERG recordings) and/or to impaired neural conduction along the post-retinal visual pathways (indexed by abnormal RCT). 

To exclude possible macular contribution to the neural conduction along the whole visual pathways, only FA patients with normal morpho-functional condition were enrolled. In fact, since macular morphological changes were considered as exclusion criteria (see point 2.1), all FA enrolled patients showed absence of macular dysfunction as derived from normal mfERG RADs responses (Table 1); therefore, no significant differences of mfERG RADs were observed in FA and Control groups.

We analyzed the influence of age at onset of the disease and duration of the disease on RGC neural conduction along the post-retinal visual pathways. By considering the age at the disease onset, FA patients were divided into two groups: late onset (FA1 group) with an age at the onset of disease >25 years and early onset (FA2 group) with an age at the onset of disease < 25 years. 

### 4.1. Neural Conduction Along the Whole Visual Pathways: VEP Data 

In all FA patients (FA group), based on descriptive and inferential statistics, o 60′ and 15′ VEP ITs and As were significantly (*p* < 0.01) increased and reduced, respectively, with respect to those of the control group.

Moreover, when the FA patients were subgrouped considering the age at the onset of disease, based on descriptive statistics, we found that FA1 group (late onset) showed significantly higher values of 60′ and 15′ VEP ITs and reduced 60′ and 15′ As with respect to FA2 group (early onset) and controls.

Our results agree with other previous studies performed in FA patients describing abnormal VEP responses [29,31,32,33,50]. The observed 60′ and 15′ VEP abnormalities suggest that in FA patients there is a dysfunction of the neural conduction along the whole visual pathway involving both large and small axons [8]. By contrast, in other previous studies considering a good association between clinical neuro-ophthalmological findings (optic nerve pallor) and VEP results, FA patients were not subdivided based on age at disease onset [1,31].

In our study, it is worth noting that the greater neural conduction dysfunction was prominent in a subgroup of FA patients in which the disease was present at a late age. However, in this group (FA1), based on inferential statistics by GLM, we were not able to establish a relationship between VEP parameters and age at onset and disease duration. 

With respect to the disease severity of FA1 patients according to SARA score, we noticed that they presented a minor disability index (SARA score between 5–13) as compared to the FA2 group. This observation suggests that in late-onset FA, predominant and more severe visual pathway involvement occurs compared to other systems. This aspect has never been previously described and needs to be confirmed by further studies. 

With respect to the aim of our work, the contribution of RGC and of the post-retinal structures in the VEP abnormalities will be discussed separately in the following Section 4.2 and Section 4.3.

### 4.2. RGC Function: PERG Data

In this study, we evaluated the RGC function by PERG recording [6,8]. By descriptive and inferential statistics, we observed that only 60′ PERG A was significantly reduced in the FA group and in the FA1 and FA2 Groups.

All this suggests that in both FA late- or early-onset groups, an RGC dysfunction can be identified.

In previous literature, few and incomplete investigations using PERG are reported in FA [29,30]. In agreement with our results, only Pinto et al. [29] observed a significant reduction of PERG A considering a single group of FA eyes with optic nerve pallor. 

The observed RGC dysfunction can be supported by an experimental model of cultured rat RGC [51]. In this study [51], by inducing metal chelation on cultured rat RGC mimicking the supposed mechanism leading to an optic neuropathy occurring in FA, an increase in RGC death secondary to oxidative damage was observed. This [51] and other findings [52,53] suggest that an abnormal regulation of intracellular iron levels due to a decreased expression of frataxin could enhance vulnerability of RGC to oxidative stress with consequent dysfunction or death.

### 4.3. Post-Retinal Neural Conduction: RCT Data

Based on the aim of our study, we assessed the neural conduction along the post-retinal visual pathways by measuring an electrophysiological index derived from the difference between VEP P100 and PERG P50 ITs that is called RCT [13,19].

To our knowledge, this is the first study that evaluates the post-retinal neural conduction in FA. In the FA1 and FA2 groups, the descriptive and inferential statistics showed that both 60′ and 15′ RCTs were significantly (*p* < 0.01) increased with respect to those of the control group; 

It is well known that the post-retinal neural conduction (indexed by RCT) may be influenced by several factors, such as the RGC function, the morpho-functional condition of the optic nerve and of the other structures forming the post-retinal visual pathways, the synaptic connectivity between the ganglion cell axons and the Lateral Geniculate Nucleus (LGN) and pathological conditions inducing demyelinating process in the visual pathways fibers [12,13,19].

About the role of RGC function on RCT, in contrast with all that occurs in glaucomatous patients in which the impaired neural conduction along the post-retinal visual pathways is related to the RGC dysfunction (the reduced PERG As are correlated to the increase of RCT) [12], in this study, we were not able to discriminate whether abnormal RCT values were sustained by impaired neural conduction along both large and small axons forming the post-retinal visual pathways (delayed RCT by using both 60′ and 15′ of visual stimulation) or by RGC dysfunction or both.

In the genesis of the observed delayed RCT, the demyelinating process that may occur in FA patients should also be considered. This is driven by the evidence observed in other electrophysiological studies in FA in which functional changes occurring in auditory and somatosensory pathways were ascribed to a demyelinating process [50,54,55,56]. 

### 4.4. Effect of BCVA, Age, Age at Onset of the Disease and Duration of the Disease in FA

In this study, we observed a significant reduction of BCVA in the FA group compared to the control group. GLM confirmed a significant group factor when FA patients were subgrouped into FA1 and FA2. Other previous studies observed that in FA patients, the reduced visual acuity occurs in later stages of the disease, generally due to optic atrophy [1,2,3,4,5,6,7,8,9,10,11,12,13,14,15,16,17,18,19,20,21,22,23,24,25,26,27,28,29,30,31,32,33,34]; however, they did not consider the subgrouping of early and late onset. In our study, those FA patients with late onset of the disease (FA1 group) showed reduced mean BCVA as compared to FA early onset (FA2 group) and controls.

Based on SARA score, FA1 patients are those with systemically less severe disease. However, the same FA1 eyes presented the worst visual acuity as compared to controls and to FA2 groups. Overall, this could suggest an effect of the onset of the disease (late onset, worse visual dysfunction) mainly on the visual system rather than other systems.

By contrast, by GLM analysis, we were not able to identify any significant relationship between electrophysiological parameters and patients’ age. All this led us to believe that, in agreement with other reports [28,31,32,50], the impairment of the whole post-retinal visual pathways is independent of the age of FA patients.

It is worth noting that in our FA1 and FA2 Groups, the GLM confirmed the absence of any significant effect of the age of onset of the disease and the disease duration on the electrophysiological parameters. We can only disclaim a slight significant effect of the duration of the disease on 15′ RCT only in the FA1 group. 

It is worth noting that since FA1 and FA2 patients were analyzed with a similar age, FA1 patients had a greater age of onset of disease than FA2 patients, but a shorter duration of disease. Thus, all that was observed in FA1 patients suggests that the impaired post-retinal conduction along the small fibers is influenced by the shorter duration of the disease only when there is a late onset of disease.

### 4.5. Study Limitations

Study limitations need to be disclaimed.

First, we included in the analysis a small number of patients, and this is due to the rarity of this neurodegenerative disorder and to the strict inclusion criteria, for which FA patients affected by ocular (macular or optic nerve) or systemic diseases that may influence the neural conduction along the visual pathways were excluded from the study. Therefore, our results must take into account the above—mentioned conditions that we applied for patient enrollment. Since in FA patients it is likely that the presence of several conditions considered as exclusion criteria (our 14 patients were selected from 49 screened patients), our results cannot be extended to the entire FA population. This also represents a remarkable limitation of the study.

Secondly, we focused exclusively on the assessment of the bioelectrical RGC responses and the neural conduction along the post-retinal visual pathways by using electrophysiological tools (simultaneous PERG and VEP recordings). Taking into account this aim, in this work, the morphological condition of the optic nerve measured by the RNFL thickness through OCT, as done in other previous reports [1,34,49,50,51,52,53,54,55,56,57,58,59], was not performed. It would be very interesting to assess whether the observed PERG and VEP abnormalities in our FA-selected patients should be associated or not with a reduction of RNFL thickness. This represents another important limitation of the study. Further studies, clarifying whether in FA patients the observed impairment is correlated to optic nerve fibers structural involvement, are required.

## 5. Conclusions

In our cohort of FA patients, selected based on specific inclusion criteria, RGC function and neural conduction along the post-retinal visual pathways are impaired. The abnormal neuronal conduction was greater in late-onset FA patients. This dysfunction is not related to patients’ age or to the age at the onset of the disease. The duration of the disease has a slight effect on the post-retinal neural conduction of small optic nerve fibers in this group.

## Figures and Tables

**Table 1:** Individual values of patients’ age, duration of disease, age of onset of the disease, best-corrected visual acuity (BCVA), multifocal electroretinogram (mfERG), pattern eletroctroretinogram (PERG) and visual evoked potentials (VEP) parameters observed in late-onset (age at the onset of disease > 25 years, FA1) and early-onset (age at the onset of disease < 25 years, FA2) Friedreich’s ataxia patients.

	Age	DD ^a^	OD ^b^	BCVA	mfERG					60′ ^c^ PERG		60′ ^c^ VEP			15′ ^d^ PERG		15′ ^d^ VEP		
	Ys ^e^	Ys ^e^	Ys ^e^	LogMAR ^f^	R1 ^g^	R2 ^g^	R3 ^g^	R4 ^g^	R5 ^g^	P50 IT ^h^ (msec) ^o^	P50-N95 A ^i^ (µV) ^p^	P100 IT ^h^ (msec) ^o^	N75-P100 A ^i^ (µV) ^p^	RCT ^l^ (msec)^o^	P50 IT ^h^ (msec) ^o^	P50-N95 A ^i^ (µV) ^p^	P100 IT ^h^ (msec) ^o^	N75-P100 A ^i^ (µV) ^p^	RCT ^l^ (msec) ^o^
					**RAD ^m^ (nV/deg^2^ ) ^n^**														
FA1#1	28	2	26	0.2	77.1	41.1	24.2	16.6	15.3	51	2.9	131	3.4	80	52	1.9	120	8.0	68
FA1#2	32	7	25	0.7	77.9	39.7	30.2	17.2	13.1	49	2.5	136	1.8	87	54	1.6	136	4.6	82
FA1#3	48	7	41	0.3	84.2	41.6	24.7	16.9	13.8	52	2.7	110	6.4	58	53	2.1	122	3.4	69
FA1#4	45	7	38	0.4	90.3	47.4	27.8	15.9	18.2	52	0.8	113	2.4	65	53	2.1	124	3.6	76
FA1#5	48	7	41	0.3	84.2	41.6	24.7	16.9	13.8	52	2.7	110	6.4	58	53	2.1	122	3.4	69
FA1#6	37	7	30	0.4	86.0	40.0	25.2	16.5	15.1	53	0.8	120	3.5	67	52	2.6	127	5.1	75
FA1#7	38	6	32	0.5	87.0	42.2	24.5	16.7	14.9	48	4.3	123	3.3	75	51	2.5	125	4.8	74
FA2#1	18	7	11	0.0	92.5	39.1	26.7	16.7	14.1	51	2.9	106	10.5	55	53	3.6	116	3.6	63
FA2#2	40	26	14	0.0	72.7	36.5	30.4	17.2	15.4	48	2.7	111	5.8	63	56	2.8	107	5.8	51
FA2#3	45	31	14	0.0	73.6	35.2	27.5	18.2	16.1	51	2.7	108	9.5	57	52	1.9	118	8.1	66
FA2#4	42	28	14	0.0	77.1	37.2	23.8	17.7	17.1	50	3.2	106	12	56	47	3.1	101	7.2	54
FA2#5	43	24	16	0.0	69.9	44.6	27.9	16.8	13.6	48	2.9	102	9.8	54	54	2.8	111	6.7	57
FA2#6	38	22	16	0.0	75.0	38.0	26.5	16.9	16.8	52	2.8	107	7.8	55	57	3.0	112	6.1	55
FA2#7	39	23	16	0.0	74.0	38.6	25.8	17.1	15.4	55	2.6	105	8.9	50	57	2.9	113	6.9	56

^a^ DD = Duration of disease; ^b^ OD = age at onset of disease; ^c^ 60′ = visual stimuli in which each check subtended 60 min of the visual arc; ^d^ 15′ = visual stimuli in which each check subtended 15 min of the visual arc; ^e^ ys = years; ^f^ LogMAR = logarithm of the Minimum Angle of Resolution; ^g^ R1, R2, R3, R4, R5 = concentric annular areas (Rings) centered on the fovea, R1 refers to 5° radius circular area, R2 refers to annular area enclosed between 5° and 10° centered on the fovea, R3 refers to annular area enclosed between 10° and 15° centered on the fovea, R4 refers to annular area enclosed between 15° and 20° centered on the fovea and R5 refers to annular area enclosed between 20° and 25° centered on the fovea; ^h^ IT = Implicit time; ^i^ A = Amplitude; ^l^ RCT = Retino-Cortical Time, difference between VEP P100 and PERG P50 Implicit Times; ^m^ RAD = Response Amplitude Density; ^n^ nV/deg^2^ = nanoVolt/degree^2^; ^o^ msec = milliseconds; ^p^ µV = microVolt.

**Table 2:** Mean values and standard deviation of multifocal electroretinogram (mfERG). Pattern eletroctroretinogram (PERG) and visual evoked potentials (VEP) parameters observed in control and Friedreich’s ataxia (FA) groups. Descriptive (mean, SD), inferential statistics R^2^ general linear model (GLM) and group factor effect.

	Control Group Mean; SD ^b^	FA Group Mean; SD ^b^	GLM ^a^ R^2^ Model % ^a^	Group Factor F; *p*
mfERG R1 ^c^ RAD ^d^ (nV/deg^2^) ^e^	77.47; 17.98	79.69; 7.29	13.43	1.40; 0.245
mfERG R2 ^c^ RAD ^d^ (nV/deg^2^) ^e^	38.40; 9.08	40.29; 3.81	4.41	1.32; 0.260
mfERG R3 ^c^ RAD ^d^ (nV/deg^2^) ^e^	25.80; 6.36	26.01; 1.91	2.58	0.14; 0.716
mfERG R4 ^c^ RAD ^d^ (nV/deg^2^) ^e^	16.85; 3.32	16.83; 0.59	9.21	0.23; 0.635
mfERG R5 ^c^ RAD ^d^ (nV/deg^2^) ^e^	14.04; 3.01	15.28; 1.44	16.66	0.30; 0.591
60′ ^f^ PERG IT ^g^ (msec) ^h^	51.20; 2.82	51.00; 0.78	4.13	0.18; 0.671
60′ ^f^ PERG A ^i^ (µV) ^l^	2.71; 0.38	2.34; 0.92	29.41	10.65; 0.003
60′ ^f^ VEP IT ^g^ (msec) ^h^	103.65; 2.60	114.00; 11.42	59.80	42.02; <0.001
60′ ^f^ VEP A ^i^ (µV) ^l^	9.22; 1.62	6.32; 3.33	57.62	38.12; <0.001
60′ ^f^ RCT ^m^ (msec) ^h^	52.45; 3.89	62.64 11.42	55.25	35.08; <0.001
15′ ^n^ PERG IT ^g^ (msec) ^h^	53.05; 4.04	52.07; 1.44	9.08	0.73; 0.399
15′ ^n^ PERG A ^i^ (µV) ^l^	2.61; 0.54	2.54; 0.53	10.78	1.37; 0.252
15′ ^n^ VEP IT ^g^ (msec) ^h^	102.55; 2.31	118.79; 9.36	84.39	137.33; <0.001
15′ ^n^ VEP A ^i^ (µV) ^l^	8.72; 1.12	6.00; 1.74	61.41	27.09; <0.001
15′ ^n^ RCT ^m^ (msec) ^h^	49.50; 4.56	66.29; 10.17	78.11	92.62; <0.001

^a^ GLM = General linear model; ^b^ SD = one standard deviation; ^c^ R1. R2. R3. R4. R5 = concentric annular areas (Rings) centered on the fovea. R1 refers to a 5° radius circular area. R2 refers to the annular area enclosed between 5° and 10° centered on the fovea. R3 refers to the annular area enclosed between 10° and 15° centered on the fovea. R4 refers to annular area enclosed between 15° and 20° centered on the fovea and R5 refers to annular area enclosed between 20° and 25° centered on the fovea; ^d^ RAD = response amplitude density; ^e^ nV/deg^2^ = nanoVolt/degree^2^; ^f^ 60′ = visual stimuli in which each check subtended 60 min of the visual arc; ^g^ IT = Implicit time; ^h^ msec = milliseconds; ^I^ A = Amplitude; ^l^ µV = microVolt; ^m^ RCT = Retino-Cortical Time. Difference between VEP P100 and PERG P50 Implicit Times; ^n^ 15′ = visual stimuli in which each check subtended 15 min of the visual arc.

**Table 3:** Mean values and standard deviation of multifocal electroretinogram (mfERG), pattern eletroctroretinogram (PERG) and visual evoked potentials (VEP) parameters observed in the control and Friedreich’s ataxia patients separate groups based on the age at onset of disease: late-onset (age at the onset of the disease > 25 years, FA1 group) and early-onset (age at the onset of disease < 25 years, FA2 group). Descriptive (mean, SD), inferential statistics R^2^ General linear model (GLM) and group factor effect.

	Control Group Mean; SD^a^	FA1 Group Mean; SD ^a^	FA2 Group Mean; SD ^a^	R^2^ (%) Model	F; *p*
mfERG R1 ^b^ RAD ^c^ (nV/deg^2^) ^d^	77.47; 17.98	82.97; 5.88	76.40; 7.43	13.91	0.76; 0.475
mfERG R2 ^b^ RAD ^c^ (nV/deg^2^) ^d^	38.40; 9.08	42.13; 3.81	38.46; 3.01	4.91	0.72; 0.497
mfERG R3 ^b^ RAD ^c^ (nV/deg^2^) ^d^	25.80; 6.36	25.08; 1.32	26.94; 2.03	3.78	0.25; 0.782
mfERG R4 ^b^ RAD ^c^ (nV/deg^2^) ^d^	16.85; 3.32	16.43; 0.32	17.23; 0.54	11.12	0.42; 0.658
mfERG R5^b^ RAD^c^ (nV/deg^2^)^d^	14.04; 3.01	15.06; 1.63	15.50; 1.30	17.39	0.27; 0.763
60′ ^e^ PERG IT^f^ (msec) ^g^	51.20; 2.82	50.71; 0.76	51.29; 0.76	1.53	0.16; 0.85
60′ ^e^ PERG A^h^ (µV) ^i^	2.71; 0.38	1.91; 1.16	2.76; 0.25	25.67	4.90; 0.015
60′ ^e^ VEP IT^f^ (msec) ^g^	103.65; 2.60	121.71; 10.61	106.29; 2.81	67.71	29.79; <0.001
60′ ^e^ VEP A^h^ (µV) ^i^	9.22; 1.62	3.46; 0.97	9.19; 1.98	70.07	33.26; <0.001
60′ ^e^ RCT^l^ (msec) ^g^	52.45; 3.89	70.00; 11.02	55.29; 5.91	58.85	20.50; <0.001
15′ ^m^ PERG IT ^f^ (msec) ^g^	53.05; 4.04	51.43; 1.51	52.71; 1.11	11.04	1.07; 0.357
15′ ^m^ PERG A ^h^ (µV) ^i^	2.61; 0.54	2.21; 0.30	2.87; 0.51	25.94	3.93; 0.031
15′ ^m^ VEP IT ^f^ (msec) ^g^	102.55; 2.31	126.43; 4.58	111.14; 5.70	87.89	93.32; <0.001
15′ ^m^ VEP A ^h^ (µV) ^i^	8.72; 1.12	5.66; 2.07	6.34; 1.42	64.35	17.83; <0.001
15′ ^m^ RCT ^l^ (msec) ^g^	49.50; 4.56	75.14; 3.67	57.43; 5.26	84.35	71.29; <0.001

^a^ SD = one standard deviation; ^b^ R1, R2, R3, R4, R5 = concentric annular areas (Rings) centered on the fovea, R1 refers to 5° radius circular area, R2 refers to annular area enclosed between 5° and 10° centered on the fovea, R3 refers to annular area enclosed between 10° and 15° centered on the fovea, R4 refers to annular area enclosed between 15° and 20° centered on the fovea and R5 refers to annular area enclosed between 20° and 25° centered on the fovea; ^c^ RAD = response amplitude density; ^d^ nV/deg^2^ = nanoVolt/degree^2^; ^e^ 60′ = visual stimuli in which each check subtended 60 min of the visual arc; ^f^ IT = Implicit time; ^g^ msec = milliseconds; ^h^ A = Amplitude; ^i^ µV = microVolt; ^l^ RCT = Retino-Cortical Time, difference between VEP P100 and PERG P50 Implicit Times; ^m^ 15′ = visual stimuli in which each check subtended 15 min of the visual arc.

**Table 4:** General linear model, F, *p* values of each covariate age at the onset of disease and disease duration for each dependent variable Visual Evoked Potentials (VEP) and Retino-cortical Time (RCT) observed in Friedreich’s ataxia patients Groups FA1 (age at the onset of disease > 25 years) and FA2 (age at the onset of disease < 25 years), respectively.

	FA1 Group R^2^ (%); F; *p*	FA2 Group R^2^ (%); F; *p*
60′ ^a^ VEP IT ^b^: age at onset of disease	52.81; 1.80; 0.282	93.31; 9.89; 0.052
60′ ^a^ VEP IT ^b^: duration of disease	52.81; 0.15; 0.727	93.31; 29.13; 0.012
60′ ^a^ RCT ^c^: age at onset of disease	29.54; 0.50; 0.530	93.10; 15.70; 0.029
60′ ^a^ RCT ^c^: duration of disease	29.54; 0.01; 0.947	93.10; 3.06; 0.179
15’ ^d^ VEP IT ^b^: age at onset of disease	74.44; 3.20; 0.172	12.42; 0.00; 0.998
15’ ^d^ VEP IT ^b^: duration of disease	7.40; 0.073	12.42; 0.37; 0.584
15’ ^d^ RCT ^c^: age at onset of disease	0.86; 0.423	35.41; 0.69; 0.467
15’ ^d^ RCT ^c^: duration of disease	88.88; 23.78; 0.013	0.40; 0.570

^a^ 60’ = visual stimuli in which each check subtended 60 min of the visual arc; ^b^ IT = Implicit time; ^c^ RCT = Retino-Cortical Time, difference between VEP P100 and PERG P50 Implicit Times; ^d^ 15’ = visual stimuli in which each check subtended 15 min of the visual arc.

## Data Availability

Data supporting reported results are available upon request to the corresponding author.

## Abbreviations

A	Amplitude
BCVA	Best-corrected visual acuity
CL	Confidence limits
DD	Duration of disease
Deg	Degree
ETDRS	Early Treatment Diabetic Retinopathy Study
FA	Friedreich Ataxia
GLM	General linear model
ISCEV	International Society for Clinical Electrophysiology of Vision
IT	Implicit Time
LGN	Lateral geniculate nucleus
LMAR	Logarithm of the Minimum Angle of Resolution
MD	Mean deviation
mfERG	Multifocal electroretinogram
mfPhNR	Multifocal Photopic Negative Response
msec	millisecond
N	Number of eyes of each group
OD	Age at onset of disease
PERG	Pattern electroretinogram
PhNR	Photopic Negative Response
RAD	Response amplitude density
RCT	Retino-cortical Time
RGC	Retinal ganglion cells
RNFL	Retinal nerve fiber layer
SD	One standard deviation of the mean
SD-OCT	Spectral domain–optical coherence tomography
SNR	Signal-to-noise ratio
V	Volt
VEP	Visual evoked potentials
